# Extending the scope of dispersive liquid–liquid microextraction for trace analysis of 3-methyl-1,2,3-butanetricarboxylic acid in atmospheric aerosols leading to the discovery of iron(III) complexes

**DOI:** 10.1007/s00216-019-01741-1

**Published:** 2019-04-01

**Authors:** Hafiz Abdul Azeem, Teshome Tolcha, Petter Ekman Hyberg, Sofia Essén, Kristina Stenström, Erik Swietlicki, Margareta Sandahl

**Affiliations:** 1 Department of Chemistry, Center for Analysis and Synthesis, Lund University, P.O. Box 124, 221 00 Lund, Sweden; 2 Department of Chemistry, Addis Ababa University, 1000 Addis Ababa, Ethiopia; 3 Department of Physics, Division of Nuclear Physics, Lund University, Box 118, 221 00 Lund, Sweden

**Keywords:** Biogenic secondary organic aerosol, MBTCA, Dispersive liquid–liquid microextraction, Metal complexes, Trace analysis

## Abstract

3-Methyl-1,2,3-butanetricarboxylic acid (MBTCA) is a secondary organic aerosol and can be used as a unique emission marker of biogenic emissions of monoterpenes. Seasonal variations and differences in vegetation cover around the world may lead to low atmospheric MBTCA concentrations, in many cases too low to be measured. Hence, an important tool to quantify the contribution of terrestrial vegetation to the loading of secondary organic aerosol may be compromised. To meet this challenge, a dispersive liquid–liquid microextraction (DLLME) method, known for the extraction of hydrophobic compounds, was extended to the extraction of polar organic compounds like MBTCA without compromising the efficiency of the method. The extraction solvent was fine-tuned using tri-*n*-octyl phosphine oxide as additive. A multivariate experimental design was applied for deeper understanding of significant variables and interactions between them. The optimum extraction conditions included 1-octanol with 15% tri-*n*-octyl phosphine oxide (w/w) as extraction solvent, methanol as dispersive solvent, 25% NaCl dissolved in 5 mL sample (w/w) acidified to pH 2 using HNO_3_, and extraction time of 15 min. A limit of detection of 0.12 pg/m^3^ in air was achieved. Furthermore, unique complexation behavior of MBTCA with iron(III) was found when analyzed with ultra-high-performance liquid chromatography coupled with electrospray ionization–quadrupole time-of-flight mass spectrometry (UHPLC–ESI–QToF). A comprehensive overview of this complexation behavior of MBTCA was examined with systematically designed experiments. This newly discovered behavior of MBTCA will be of interest for further research on organometallic photooxidation chemistry of atmospheric aerosols.

**Electronic supplementary material:**

The online version of this article (10.1007/s00216-019-01741-1) contains supplementary material, which is available to authorized users.

**Graphical abstract:**
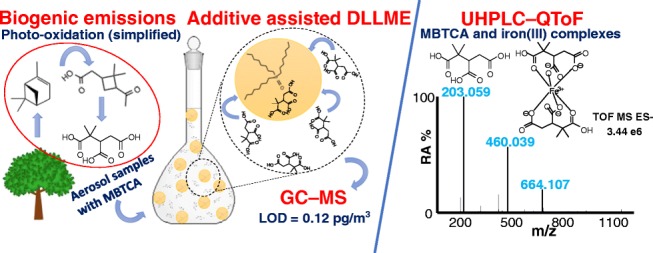
**a**) Additive assisted DLLME and MBTCA complexes with Fe(III), **b**) A good quality figure is attached in ppt format to facilitate editable objects

## Introduction

Monoterpenes are a class of biogenic compounds that are well known for their high emission rates of up to 127 Tg per year on the global scale [1]. Photooxidation of monoterpene emissions like α-pinene in the atmosphere produces 3-methyl-1,2,3-butanetricarboxylic acid (MBTCA) [2]. As MBTCA is a unique photooxidation product of α/β-pinene, it serves as an emission marker for biogenic emissions coming from monoterpenes [3]. Kanakidou et al. [4] and Szmigielski et al. [5] further highlighted that the study of MBTCA could help understand the complex chemistry of biogenic secondary organic aerosols in the atmosphere. Estimation of MBTCA can provide more information on the relative contribution of anthropogenic versus biogenic emissions to the atmospheric aerosol loading.

MBTCA concentrations are often too low to be quantified using regular sample preparation techniques such as ultrasonic assisted liquid–liquid extraction [2]. Most of the studies reporting MBTCA are based on samples collected by high volume air samplers that filter a large quantity of air through a membrane/filter [6]. Several sampling stations are equipped with medium or low volume air samplers that are typically unable to sample sufficient amounts of aerosol particle mass to enable subsequent MBTCA analysis. In addition, it is challenging to estimate the whole suite of emissions that contribute to the organic aerosol, including both anthropogenic and biogenic emissions in the regions with limited vegetation, e.g., Australia, Middle East, and Africa. Kourtchev et al. [2] reported that the concentrations of MBTCA were below their limit of detection (LOD) in autumn and winter samples collected for 48 h using a flow rate of 500 L/min. The emissions of α/β-pinene are strongly linked to photosynthesis and temperature, and are significantly lower during colder periods compared to summer, in particular for coniferous trees [7]. This further adds to the challenge of performing quantitative analysis of MBTCA in aerosol samples throughout the year. It is therefore important to develop more efficient sample preparation and analytical methods to reach lower LOD of MBTCA.

Dispersive liquid–liquid microextraction (DLLME) has gained popularity owing to its simplicity, low cost, enrichment of analyte, and short extraction times [8–12]. However, traditional sample preconcentration techniques like solid-phase extraction have been used for the extraction of polar compounds from aerosols [13]. DLLME has long been used for non-polar moieties only. Few attempts have been made to extract polar organic compounds and metal ions by ion-pairing and complex formation prior to DLLME [14, 15]. The extraction efficiency in such methods is, however, largely dependent on the atom economy of pre-DLLME reactions. In some recent studies, Faraji et al. [16] and Ma et al. [17] reported the extraction of halogenated compounds like trihalomethanes and haloacetonitriles using 1-dodecanol and dichloromethane as extraction solvents, respectively. On et al. [18] extracted halomethanes and haloacetonitriles using dichloromethane as the extraction solvent. However, MBTCA being a highly polar compound (log* P* = − 0.3) as compared to halogenated methanes and acetonitriles (log *P* = 0.29–2.16) may require a water-immiscible extraction solvent with highly polar characteristics. The present study attempts to fine-tune the chemistry of extraction solvent by introducing a single-step additive-assisted DLLME for the extraction of polar organic acids, such as MBTCA. Contrary to the commonly used ultrasonic assisted extraction of aerosol samples using several milliliters of mixtures of hazardous organic solvents like dichloromethane, methanol, hexane [2, 6, 19], additive-assisted DLLME is more selective and green owing to the possibility of fine-tuning the chemistry of extraction solvent with suitable additives and reduced use of organic solvents, respectively. This new dimension of DLLME provides efficiency comparable to traditional DLLME and the lowest LOD of MBTCA in aerosols (picograms per cubic meter) by DLLME and GC–MS reported so far.

In an effort to lower the limit of detection of MBTCA, we compared GC–MS data with ultra-high-performance liquid chromatography coupled with electrosprayionization–quadrupole time-of-flight mass spectrometry (UHPLC–ESI–QToF) and discovered signal splitting in ESI–MS that indicated a possible bias in the estimation of MBTCA using UHPLC–ESI–QToF (see Electronic Supplementary Material (ESM) Fig. S1). Further investigations by UHPLC–ESI–QToF led to the discovery of an interesting metal complexation behavior of MBTCA. Although metal complexes of organic acids in environmental soil samples are known [20], little is known about the formation and existence of such complexes in complicated atmospheric photooxidation chemistry. Kostenidou et al. [21] presented a study dedicated to the physical and chemical properties of MBTCA. Myllys et al. [22] and Aljawhary et al. [23] reported cluster formation of MBTCA molecules. To the best of our knowledge, this is the first time a comprehensive study is presented on complexation behavior of MBTCA. Discovery of metal complexes of MBTCA can open up new research questions on the chemical properties of MBTCA, lifetime of its metal complexes, and the chemistry of its interactions with other substances in atmospheric aerosols.

## Materials and methods

### Chemicals and standards

LCMS-grade methanol was purchased from Honeywell, Seelze, Germany. NaHCO_3_ (99%), NH_4_CO_3_ (analytical grade), dihexylether (97%), methyl dodecanate (99%), octanol (HPLC-grade, 99%), heptane (99% HPLC-grade), and* N*,*O*-bis(trimethylsilyl)trifluoroacetamide (BSTFA, GC-grade and 98% excluding trimethylsilyl chloride) containing 1% trimethylsilyl chloride (TMCS) were purchased from Sigma-Aldrich, Munich, Germany. *n*-Hexane (96%, analytical grade) and ammonia (25%, reagent grade) were purchased by Scharlau, Sentmenat, Spain. Tri-*n*-octyl phosphine oxide (TOPO, 99%) and 1-phenyldodecane (97%) were purchased from Acros, Geel, Belgium. 3-Methyl-1,2,3-butanetricarboxylic acid (MBTCA) was purchased from Toronto Research Chemicals Inc., Toronto, Canada. HNO_3_ was purchased from Merck, Darmstadt, Germany. Helium gas (5.0) was purchased from AGA Gas AB, Malmö, Sweden. MilliQ water purified by a Q-Gard® 1 (Millipore, Bedford, MA, USA) equipped with a 0.22-μm Millipak® Express 20 filter.

### Aerosol sampling

Aerosol sampling was conducted at the ACTRIS (Aerosols, Clouds, and Trace gases Research InfraStructure Network) station situated at Vavihill in southern Sweden (56°01′ N, 13°09′ E, 172 m a.s.l.). A detailed description of sampling is given elsewhere [24–26]. Briefly, PM_10_ samples were collected on 47-mm quartz-fiber filters (Pallflex 2500QAT-UP) for 72 h at a flow rate of 38 L/min using a Leckel SEQ47/50 sampler. Filters were baked at 900 °C for 4 h before sampling. After sampling the filters where placed in petri dishes wrapped in aluminum foil and packed in ziplock bags. Samples were stored in a freezer at −18 °C until analysis. Aerosol filter samples were cut into small pieces and MBTCA was extracted into 5 mL milliQ water acidified to pH 2 by HNO_3_ by ultrasonication for 20 min. The extract was filtered with a 0.45-μm polypropylene membrane syringe filter and used for DLLME.

### Screening of qualitative variables for DLLME

Three qualitative variables, i.e., type of extraction solvent, presence of TOPO as additive in the extraction solvent, and type of dispersive solvent were investigated. For the initial optimization, methyl decanoate, 1-octanol, 1-hexanol, and dihexyl ether were tested as extraction solvents. 1-Hexanol and dihexyl ether were found to be unsuitable because of partial solubility in water. Methyl decanoate and 1-octanol were further evaluated with and without the presence of TOPO in 1-octanol, up to 15% TOPO (w/w) because of limited solubility, and tested with all other experimental conditions kept constant. Methanol was used both as dispersive solvent and as de-emulsifying agent (500 μL each) to break the emulsion when extraction is completed, and 10% NaCl (w/w) dissolved in standard solutions was used for extractions. Standard solutions of 5 mL milliQ water, acidified to pH 2.5 by HNO_3_ and spiked to 1 μg/mL MBTCA, were used for extractions. Extractions were performed during 5 min. Six organic solvents, i.e., acetonitrile, acetone, 1,4-butanediol, diethanolamine, methanol, and 2-propanol, were screened as dispersive solvents. In addition to qualitative variables, screening of two quantitative variables, i.e., pH of sample and volume of dispersive solvent, was performed to find suitable ranges for further optimization. Standard MBTCA solutions acidified to pH 1, 2, 3, and 4 were extracted by DLLME. Three different volumes of dispersive solvent, i.e., 50, 100, and 150 μL, were used to narrow down the range of suitable volume of extraction solvent for further optimization of the method.

### Multivariate design of experiment for DLLME

A central composite faced design was used to optimize independent quantitative variables including extraction time, amount of salt, volume of extraction solvent, and volume of dispersive solvent. Software package MODDE 10.1.1 (Umetrics, Umeå, Sweden) was used for calculations and evaluation of the results. Samples were analyzed by both UHPLC–ESI–QToF and GC–MS (method validation data is presented from GC–MS experiments).

### Analysis of aerosols samples

Aerosol filter samples were cut into small pieces and extracted into 5 mL milliQ water acidified to pH 2 by HNO_3_ by ultrasonic extraction for 1 h. The extracts were filtered using a 0.45-μm polypropylene membrane syringe filter followed by DLLME. The derivatization and GC–MS methods were performed according to our earlier study [26] with some modifications (see ESM).

### Complexation behavior in UHPLC–ESI–QToF

Complexation behavior of MBTCA was observed in UHPLC–ESI–QToF. UHPLC–ESI–QToF was used initially to study the performance of DLLME in parallel to the GC–MS method. However, after the discovery of MBTCA complexation with iron(III), UHPLC–ESI–QToF analysis was dedicated to the study of MBTCA–iron(III) complexes. The experiments were repeated several times to confirm the splitting of the signal using standard solutions of MBTCA prepared at different concentrations as well as in both positive and negative ESI modes. A brief description of UHPLC–ESI–QToF is given below*.*

Aqueous standards and extracts of MBTCA were analyzed by Waters Acquity UPLC with a Waters XEVO-G2 QToF (Waters Corporation, Milford, MA). The analysis was performed using an Acquity UPLC CSH C18 column (1 .7 μm, 2.1 × 100 mm) from Waters. The column temperature was maintained at 60 °C with a flow rate of 0.5 mL/min. The mobile phase consisted of 0.1% formic acid in both water (A) and methanol (B) with a gradient starting at 3% B, then increased from 3% to 20% B in 4 min, 20% to 95% B over 4 to 5 min, and finally returned to 3% B over 5 to 5.10 min and held until 8 min. Mass spectrometer scans were performed from* m*/*z* 50–1200 with ESI tuned between cone voltages 10–30 V and capillary voltages 1–2.5 kV in both positive and negative modes. The desolvation gas (N_2_) flow rates and temperature were 400 L/h and 500 °C, respectively. The cone gas flow rate was 50 L/h and source temperature was 120 °C. Data were acquired by Waters MassLynx 4.1 (Waters MS Technologies, Manchester, UK).

## Results and discussion

### Screening of qualitative variables for DLLME

Previous studies have shown that different types of extraction and dispersive solvents have significant effects on extraction efficiencies [9, 27]. In principle, DLLME requires an extraction solvent to be immiscible with water. A dispersive solvent miscible with both aqueous sample and extraction solvent facilitates the dispersion of extraction solvent in the sample, leading to a high surface area that enhances extraction efficiency. In this study, it is demonstrated that the addition of suitable additives into the extraction solvent can be used to extract highly polar organic acids such as MBTCA. TOPO is a well-known additive used in membrane extractions of organic acids and phenols [28, 29]. The phosphine group of TOPO interacts with polar compounds like carboxylic acids as a result of dipole interactions while long *n*-octyl chains maintain its solubility in non-polar solvents like octanol. This feature of TOPO was used in DLLME to extend the application window of the technique beyond non-polar compounds.

It was observed that the amount of MBTCA extracted increased with increasing amounts of TOPO added to 1-octanol (ESM Fig. S2). However, as a result of solubility limitations, a maximum soluble amount of TOPO (15%) in 1-octanol was used for further optimization. Out of six dispersive solvents used, methanol, acetonitrile, and acetone provided the highest extraction efficiencies while 1,4-butanediol, diethanolamine, and 2-propanol provided poor extractions. Methanol, acetonitrile, and acetone, being smaller molecules in size with high dipolarity, produce better dispersion of 1-octanol in water enhancing the extraction of organic acids. The amounts of MBTCA extracted by means of methanol, acetonitrile, and acetone, as dispersive solvents, were comparable without significant difference (ESM Fig. S3). Therefore methanol was chosen as the dispersive solvent owing to its greenness and compatibility with plastic containers. Enhanced extraction of MBTCA was observed at sample pH 2 or lower. This can be explained by the p*K*_a_ of MBTCA. The lowest calculated p*K*_a_ of MBTCA is 3.50 ± 0.15 [30]; MBTCA exists in protonated neutral form in the solution at pH 2 and below. Protonated MBTCA is more prone to partition to 1-octanol where TOPO helps retain the organic acid in the extraction solvent. Experiments were performed using 50, 100, and 150 μL of extraction solvent to explore an appropriate working range and 150 μL was chosen for further optimization on the basis of its highest enrichment of MBTCA.

### Multivariate experimental design

**Fig. 1:**
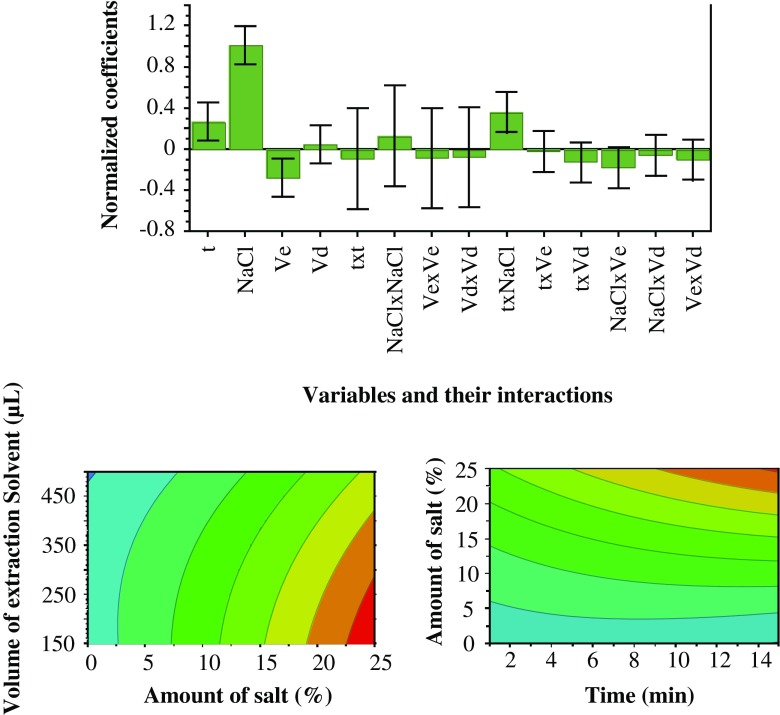
Normalized coefficient plot (top row) represents variables and their significance on response. Ve, Vd, and t represent volume of extraction solvent (1-octanol + 15% TOPO), volume of dispersive solvent (methanol), and extraction time, respectively (as the coefficients have different ranges of responses, the coefficients are normalized by dividing with standard deviation of their response. Positive values represent an increase in MBTCA extraction on higher values of a coefficient and vice versa). Contour plots (bottom row) represent amount of MBTCA extracted, blue to red transition represents minimum to maximum amount of MBTCA extracted

A central composite faced design was used to optimize the four quantitative variables: extraction time, amount of salt, volume of extraction solvent, and volume of dispersive solvent. A total explained variance of 94% [*R*^2^(*Y*) = 0.94] and a cross-validated predictability of 67% [*Q*^2^(*Y*) = 0.67] were obtained. Figure 1 (coefficient plot) shows the influence of the four qualitative variables. The amount of salt dissolved in the sample was the only statistically significant factor. Furthermore, quadratic interactions between the variables also illustrated that larger amounts of salt and longer extraction times positively influence MBTCA extractions (Fig. 1, contour plots). However, as a result of solubility limitations, 25% NaCl (m/v) was used. No investigation was performed to test different types of inorganic salts. Further experiments were performed with extraction times of 15, 20, 25, and 30 min. No significant difference was observed in extraction efficiencies between extraction times of 15 and 20 min. However, the extraction time of 25 min showed slightly higher extraction of MBTCA with poor repeatability (ESM Fig. S4). It was observed that the emulsion started to deform during longer extraction times, which explains the higher standard deviations with longer extraction times. On the basis of the results, an extraction time of 15 min was used.

### Performance of the DLLME–GC–MS method

The optimized DLLME shows low limits of detection and quantification. The obtained limit of detection of MBTCA (0.12 pg/m^3^) is significantly lower than the limits of detection reported by, e.g., Fu et al. [6], Zuth et al. [31], Ding et al. [19], and Kourtchev et al. [2] that were 0.005, 1.3, 2, and 4 ng/m^3^ respectively. Tadesse et al. [12] reported enrichment factors of 10.2–19.6 for DLLME of multiclass pesticide residues in water samples. Additive-assisted DLLME for extraction of polar compounds such as MBTCA provided an enrichment factor of 16.6 that is fairly comparable to earlier DLLME studies of non-polar compounds. This highlights the potential of additive-assisted DLLME for the extraction of polar organic acids with comparable efficiency. Intra- and interday precisions (2 μg/mL, *n* = 3) were 4.7% and 10.3%, respectively. It is worth highlighting that the relative standard deviations represent all the experimental steps from DLLME to GC–MS analysis (ESM Table S1). To show its potential, the optimized method was applied to aerosol samples collected in autumn and winter of 2011–12 at the rural site of Vavihill situated in a densely forested area. The concentrations of MBTCA were between 1.9 and 14.4 μg/m^3^, fairly higher than many ACTRIS sites situated in southern Europe (Table 1).

**Table 1:** Concentration of MBTCA (μg/m^3^) in aerosol samples collected at Vavihill sampling site

Sampling date	MBTCA (μg/m^3^)
13.07.2011	8.34
11.12.2011	2.16
17.12.2011	3.09
23.12.2011	14.37
07.01.2012	1.90

### Complexation behavior in UHPLC–ESI–QToF

**Fig. 2:**
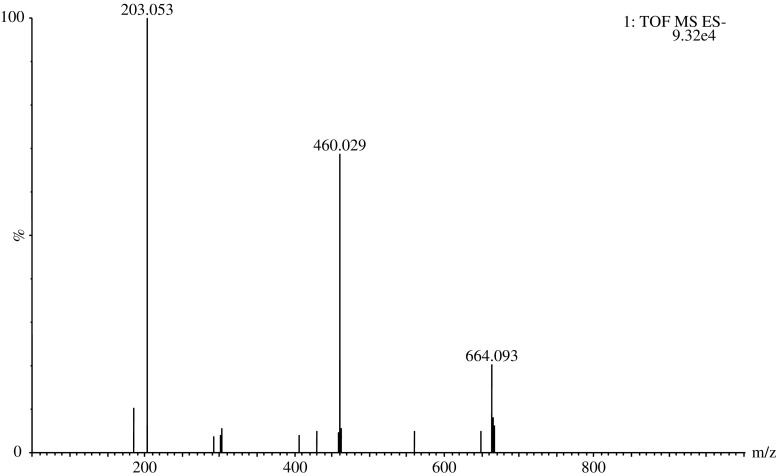
MBTCA, [2MBTCA-4H + Fe]^−^, and [3MBTCA-4H + Fe]^−^ complexes in negative ESI–MS as* m*/*z* 203.053, 460.029, and 664.093, respectively (*m*/*z* values obtained from MassLynx 4.1)

The complexation behavior of MBTCA was observed using UHPLC–ESI–QToF when injecting a standard solution of MBTCA prepared in milliQ water. Interestingly, three distinct* m*/*z* signals, i.e., 203, 460, 664 were observed in the negative mode as shown in Fig. 2 (similar behavior was also seen in the positive mode). The signal splits into ions with distinct* m*/*z*, i.e., 203, 460, and 664 assigned to MBTCA, [2MBTCA-4H + Fe]^−^, and [3MBTCA-4H + Fe]^−^ complexes, respectively.

**Fig. 3:**
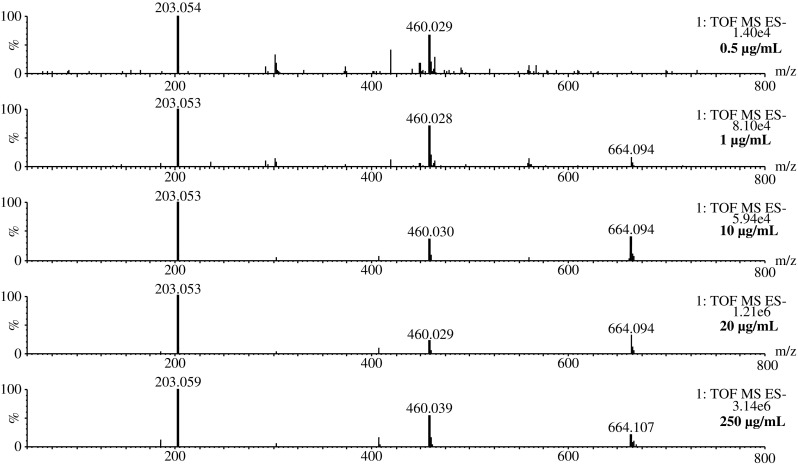
MBTCA complexes with Fe(III) in negative ESI–MS at different concentrations between 0.5 and 250 μg/mL. At low concentrations, e.g., 0.5 μg/mL, the signal of [3MBTCA-4H + Fe]^−^ (*m*/*z* 664) is still available but quantitatively insignificant because of noise

A steady complexation behavior of the analyte was observed in experiments performed using 0.5–250 μg/mL standard solutions of MBTCA (Fig. 3). Furthermore, different capillary voltages (1–2.5 kV) and cone voltages (10–30 V) were tested to investigate the role of both positive and negative electrospray ionization in the formation of iron(III) complexes. Regardless of different combinations of capillary and cone voltages, the complexes were observed in all runs. It was, however, observed that fine-tuning the voltages influences the relative abundance of the ions. Therefore, it is recommended that ESI–MS signals of both MBTCA and its subsequent complexes should be considered by UHPLC–ESI–QToF users for qualitative as well as quantitative analysis of MBTCA in aerosol samples, especially when dealing with samples of low concentrations. A simplified hypothetical pathway of complex formation and proposed structures of the complexes are given in ESM (Figs. S5 and S6).

In order to establish if the complex formation is a UHPLC–ESI–QToF phenomenon or if it also exists in atmospheric aerosols further experiments were performed. Initially, a standard solution of MBTCA was prepared with 0.1% EDTA solution (0.1 M) in milliQ water for direct infusion using negative ESI–MS. Two distinct ions, i.e.,* m*/*z* 203 and 291 representing MBTCA and EDTA, were observed (ESM Fig. S7). The same solution showed ions for EDTA and iron(III) complexes of MBTCA resolved in time in UHPLC–ESI–QToF. This indicates the presence of MBTCA complexes with iron(III) in the chromatographic system before reaching MS. In another experimental setup, all glassware were soaked in 1 M HNO_3_ overnight and rinsed with milliQ water to achieve an environment that is as iron-free as possible as iron is present everywhere in our ambient environment. A standard solution of MBTCA prepared in a so-called iron-free environment gave rise to the same iron(III) complexes in UHPLC–ESI–QToF. These results confirm that MBTCA produces metal complexes and iron(III) complexes may be avoided using EDTA in direct infusion mode. One can argue that MBTCA may interact with iron from parts of the instrument such as the ESI spray capillary; however, it also opens new research questions about the behavior of MBTCA and the possibilities of complex formation with iron(III) and other metals readily available in the atmosphere. To our knowledge, scarce information is available about the complexation behavior of organic acids and sugars in atmospheric aerosols. It is therefore expected that this discovery will help the scientific community to better understand the dynamics of chemical reactions in the atmosphere.

## Conclusion

Here we describe an extension of DLLME for the extraction of MBTCA, a polar organic acid. Additive-assisted DLLME promotes extraction of MBTCA with an enrichment factor of 16.6. The method can be used to estimate MBTCA in aerosol samples for better understanding of biogenic emissions in different seasons as well as areas of lower vegetation. We also present the discovery of a novel complexation behavior of MBTCA with iron(III). Iron being the most abundant metal in the earth’s crust and readily available in the atmosphere undergoes organo-iron complex formation as reported earlier for MBTCA. In conclusion, analytical chemists using LCMS are recommended to consider possible signal splitting in MS between MBTCA and its iron(III) complexes as the complexation behavior was observed in aerosol samples as well as standard solutions of MBTCA. Further studies are required to fully understand the interactions of MBTCA and similar organic aerosols with iron and other metals present in the atmosphere.

## Electronic supplementary material

ESM 1 (PDF 629 kb)
