# Supplementary material for: Extending the scope of dispersive liquid–liquid microextraction for trace analysis of 3-methyl-1,2,3-butanetricarboxylic acid in atmospheric aerosols leading to the discovery of iron(III) complexes
Source: Anal Bioanal Chem. 2019 Apr 1;411(13):2937–44. doi: 10.1007/s00216-019-01741-1 (PMC6522453; doi:10.1007/s00216-019-01741-1)
Supplement: Supplementary file 1 — (PDF 629 kb) [file 216_2019_1741_MOESM1_ESM.pdf]

Analytical and Bioanalytical Chemistry

Electronic Supplementary Material

Extending the scope of dispersive liquid–liquid microextraction for trace analysis of 3-methyl-1,2,3-butanetricarboxylic acid in atmospheric aerosols leading to the discovery of iron(III) complexes

Hafiz Abdul Azeem, Teshome Tolcha, Petter Ekman Hyberg, Sofia Essén, Kristina Stenström, Erik Swietlicki, Margareta Sandahl

Table of contents

Simplified flowchart of the study………...…………………………………... Page 3

Chromatographic analysis ………..………………………………………….. Page 3

Analytical performance of DLLME (Table S1) ……………………………... Page 4

Screening of extracting and dispersing solvents ……………….……………. Page 4

Complexation behavior ……………………………………...………….. Page 6

Spectrum of MBTCA/EDTA solution (Figure S7) …………………………... Page 8

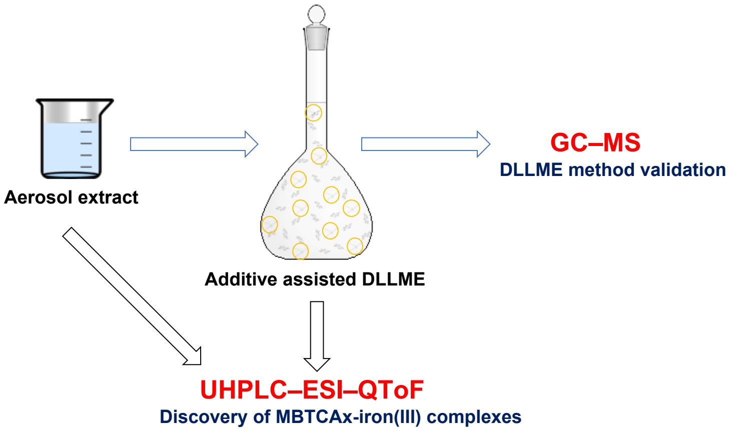

Fig. S1 A simplified flowchart of the study represents use of GC–MS for validation of DLLME whereas UHPLC–ESI–QToF for complexes of MBTCA

Chromatographic analysis

Extracting solvent from DLLME, enriched with MBTCA, was pretreated for chromatographic analysis. It was observed that TOPO (from extracting solvent) interferes in derivatization reactions (using silylation agents) for GC–MS analysis. Its high boiling point and surface tension can cost excessive cleaning of LCMS systems. Therefore, MBTCA was re-extracted from the extracting solvent using two aliquots of 150 µL of pH 9.5 buffer ($NH_3OH/(NH_3)_2CO_3$) and ultrasonicated for 15 min each, using a sonication bath (5510 Branson, Cleanosonic, Richmond VA, USA). The two aliquots were pooled together and used for further analysis.

GC–MS analysis

The extracts were evaporated with 100 µL of acetone at 40 °C under a gentle stream of N_2. Addition of a volatile aprotic solvent like acetone breaks intermolecular forces and facilitates evaporation. Then the samples were derivatized with 15 µL of hexane containing internal standard and 10 µL of N,O-Bis(trimethylsilyl)trifluoroacetamide (BSTFA) containing 1% Trimethylchlorosilane (TMCS) at 80 °C for 1 hr as described earlier by Martinsson et al. [1]. Samples were injected in GC–MS immediately after derivatization.
Samples were analyzed by Agilent 6890 series GC with 5973 MS (Agilent Technologies, Palo Alto, USA) using Agilent HP-5ms column (30 m x 0.25 mm x 0.25 µm film thickness). Chromatorgaphic analysis was performed according to our study presented earlier [1] with some modification in the oven program. An initial oven temperature of 60 °C was held for 3 min then the temperature was raised to 170 °C at a rate of 15 °C/min, finally the temperature was raised to 300 °C at a rate of 30 °C/min and held for 4 min. Derivatized MBTCA and internal standard (1-phenyldodecane) were identified and quantified by ions m/z 405 and 246, respectively.

Table S1 Analytical performance of the proposed DLLME method, LOD obtained by the comparison of S/N ratio

Linear range using standard solutions (ng/L)	2 – 10000
Co-efficient of determination (R^2)	0.99
LOD in air (pg/m^3)	0.12
Intraday precision, RSD (n=3)	4.7%
Interday precision, RSD (n=3)	10.3%

Screening of extracting and dispersing solvents

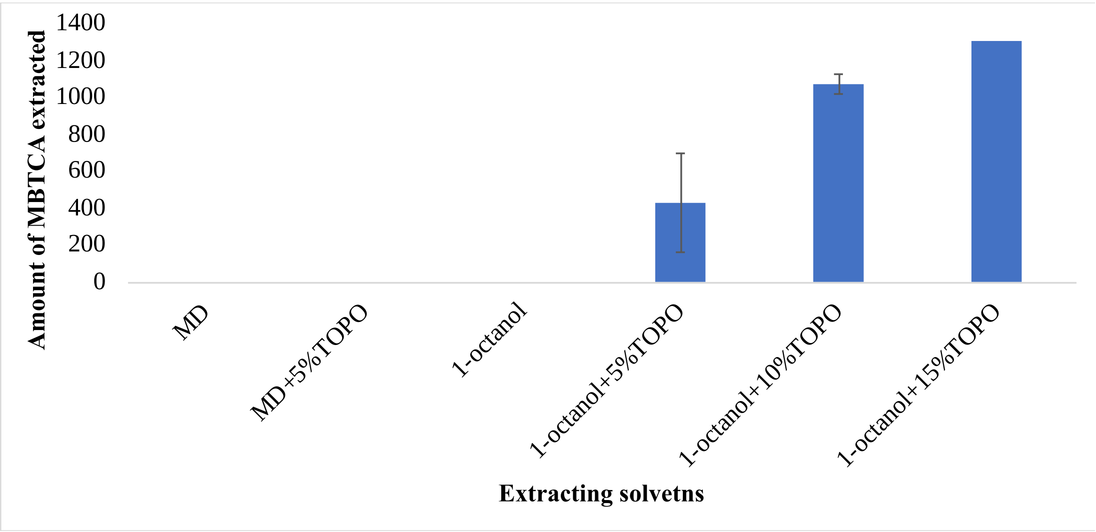

Fig. S2 The figure represents a comparison methyldecanoate and 1-octanol (as extracting solvents) with and without the addition of 5% TOPO. The experiments were furhter extended to 1-octanol with 10% and 15% TOPO (w/w). Error bars represet standard difference of dupicates. MD=methyldecanoate and TOPO=tri-*n*-octyl phosphineoxide

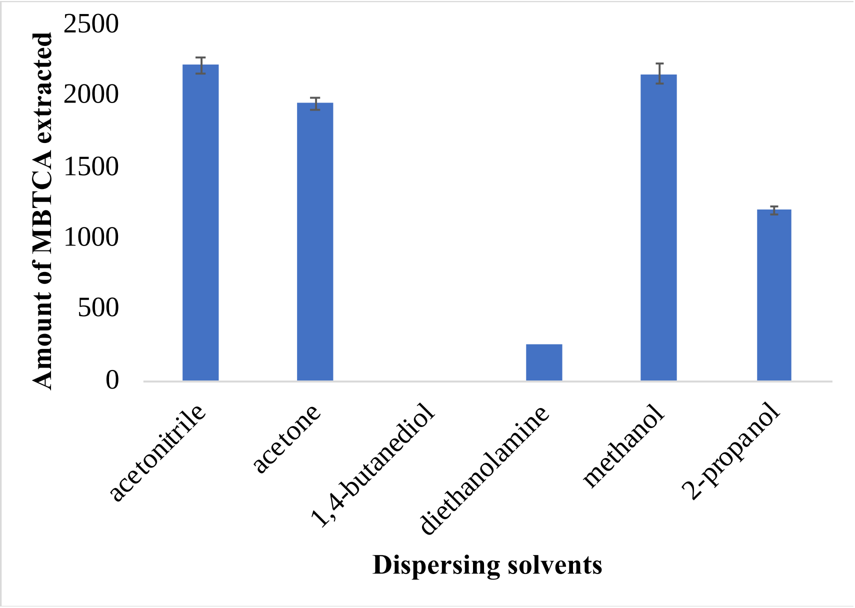

Fig. S3 A comparison of six dispersing solvents screened for the extraction of MBTCA (n=2)

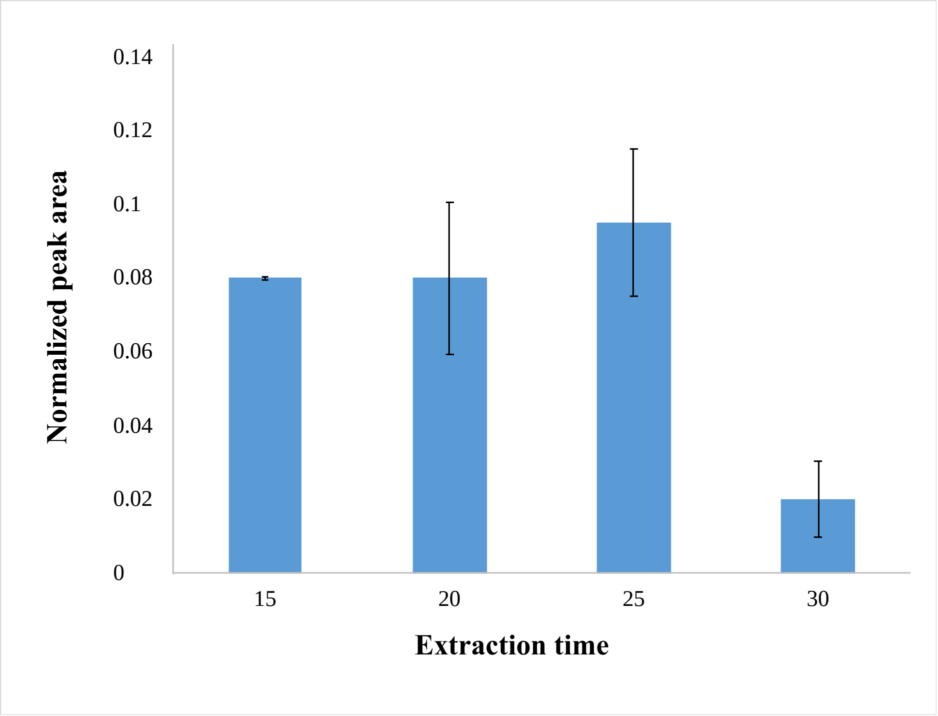

Fig. S4 DLLME extraction time extended to 15, 20, 25 and 30 min (n=2)

Complexation behavior

Due to similarities in chemical structures of MBTCA and citric acid, stability constants (log K_1) of citric acid-metal complexes were taken as a reference [2]. It can be inferred that MBTCA undergoes iron(III) complex formation. Figure S2 shows hypothetical structures of $[2MBTCA\text{-}4H+Fe]^-$ together with a possible synthesis pathway. It is noteworthy that the presented structures are drawn as closest to $[2MBTCA\text{-}4H+Fe]^-$ (Figure S3) and $[3MBTCA\text{-}4H+Fe]^-$ ions observed, and more studies are required to understand the structure and properties of MBTCA and metal complexes.

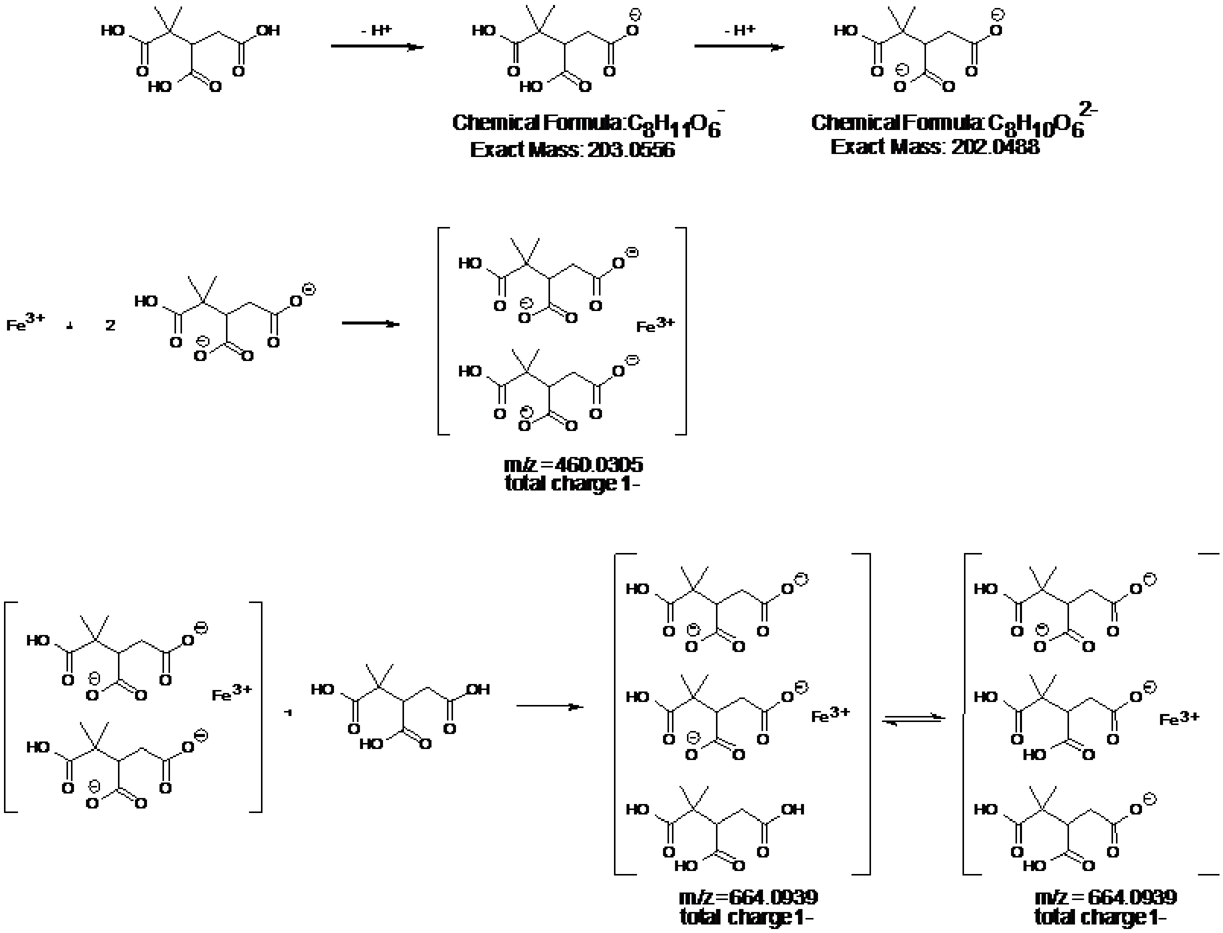

Fig. S5 Simplified hypothetical pathway of the synthesis of $[2MBTCA\text{-}4H+Fe]^-$ and $[3MBTCA\text{-}4H+Fe]^-$ from MBTCA and their theoretically calculated masses

The proposed [2MBTCA-4H+Fe]$^{-}$ is a complex of iron(III) and two double-deprotonated molecules of MBTCA containing a net charge 1-. The calculated m/z matches the measured one fairly well and reasonably explains the proposed species along with all the mentioned conditions and prior knowledge. The following structure can be proposed for [2MBTCA-4H+Fe]$^{-}$, since X-ray structural analyses often uncover that iron(III) complexes prefer an octahedral coordination (Figure S3). The observation of the third prominent peak (m/z 664.1074) may be explained in terms of being initially formed as a hydrogen-bonded suppramolecular aggregate of [2MBTCA-4H+Fe]$^{-}$ with an additional molecule of MBTCA following a mutual charge exchange in equilibrium with the other deprotonated MBTCA molecules of the complex takes place.

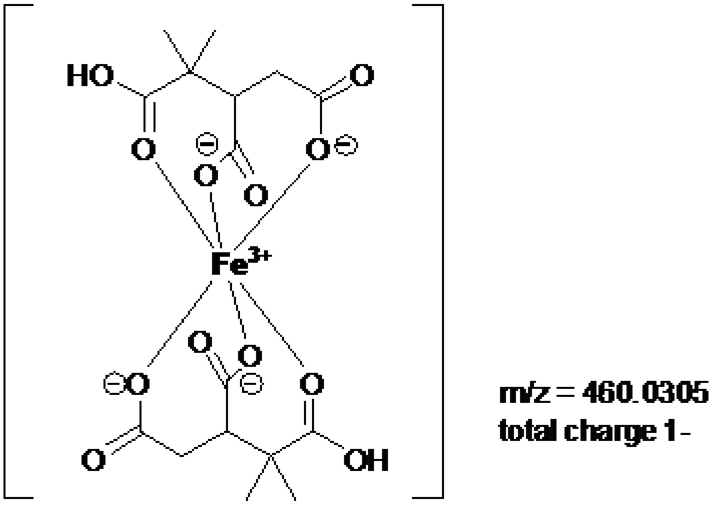

Fig. S6 An octahedral structure of [2MBTCA-4H+Fe]$^{-}$ proposed that with m/z 460.031

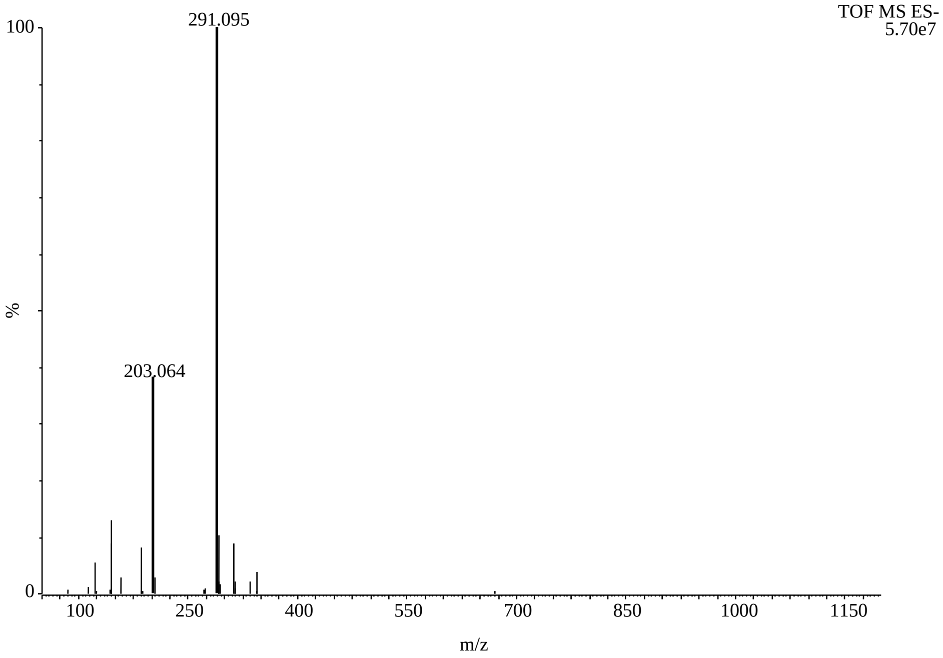

Fig. S7 Mass spectrum of standard solution of MBTCA prepared with 0.1% EDTA (0.1M) in milliQ water injected in negative ESI-MS by direct infusion. Two distinct ions i.e. m/z 203 and 291 represent MBTCA and EDTA but no iron(III) complexes

References

(1) Martinsson, J.; Azeem, H. A.; Sporre, M. K.; Bergstrom, R.; Ahlberg, E.; Ostrom, E.; Kristensson, A.; Swietlicki, E.; Stenstrom, K. E. Atmos Chem Phys 2017, *17*, 4265-4281.
(2) CRC Handbook of Food Additives. second ed. USA: CRC Press; 1972.
